# A glimpse into darkness: Diversity of culturable cyanobacteria, green algae and fungi from subaerial cave biofilms

**DOI:** 10.1111/jpy.70104

**Published:** 2025-12-10

**Authors:** Patrick Jung, Laura Briegel‐Williams, Dennis J. Nürnberg, Tobith Wolf, Antonio Guillen, Manuel Leira, Michael Lakatos

**Affiliations:** ^1^ XCEL – Extreme Cryptogam Ecology Lab University of Applied Sciences Kaiserslautern Kaiserslautern Germany; ^2^ Institute for Experimental Physics Freie Universität Berlin Berlin Germany; ^3^ Dahlem Centre of Plant Sciences Freie Universität Berlin Berlin Germany; ^4^ Astroland Agency Logroño Spain; ^5^ Parque Científico y Tecnológico de Cantabria Santander Spain; ^6^ Integrative Biotechnology University of Applied Sciences Kaiserslautern Pirmasens Germany

**Keywords:** biofilms, caves, microbial diversity, subterranean

## Abstract

Caves and hypogean environments provide stable microclimates characterized by uniform temperatures, constant humidity, and low light levels. In such sites, extremely low light irradiance can support the growth of subaerial biofilms (SABs) dominated by unique photosynthetic communities of cyanobacteria accompanied by chlorophytes, heterotrophic bacteria, and fungi. This study aimed to apply a culture‐dependent approach to uncover the diversity of cyanobacteria, green algae, and fungi from SABs of a cave in Northern Spain. We isolated a total of 58 cyanobacteria grouped into 21 genera based on their 16S rRNA gene sequences; 24 green algae grouped into 10 genera based on their SSU rRNA gene sequences, and 41 fungi fell into 13 genera according to phylogenies based on the ITS rRNA region between the 16S and 23S rRNA genes (ITS rRNA region). The SABs were dominated by cyanobacteria, which also reflected by high diversity, including calcium‐carbonate sheath‐producing species such as *Geitleria calcarea* and *Scytonema julianum.* Typical cave‐inhabiting species such as *Chalicogloea cavernicola*, *Timaviella karstica* or *Oculatella subterranea* were also isolated, alongside potentially new genera and species. Associated green algae were predominantly located closer to the cave entrance and included various lineages of the genera *Jenufa* and *Chromochloris*. The fungal community directly derived from the SABs was dominated by decomposers, saprophytes, and phytoparasitic representatives such as *Sporobolomyces*, *Stereum*, and *Phlebia*, with a corresponding set of enzymes that were evaluated for all fungal isolates. The results strongly support the hypothesis that specialized cyanobacterial communities are often located in caves as a result of niche specialization.

**Abbreviations**
BBM: Bold's Basal Medium
BI: Bayesian inference
EPS: extracellular polymeric substances
ML: maximum likelihood
PCR: polymerase chain reaction
SABs: subaerial biofilms

## INTRODUCTION

Caves exist worldwide and span a remarkable array of geological settings, most commonly forming in soluble rocks such as limestone, dolomite, and gypsum through karst processes driven by carbonic or sulfuric acid dissolution. These solutional (karst) caves represent over 95% of known cave systems, although less common volcanic (lava tube) and ice caves also contribute significantly to subterranean biodiversity (Gunn, 2004). Cave substrata may include calcite, aragonite, selenite, sulfates, and iron oxides, leading to diverse mineralogies that influence microbial habitats (Northup & Lavoie, 2001). Although most explored caves are located in Europe, North America, Asia, and Oceania, extensive unstudied systems are likely present in regions such as Africa, South America, and China, which may contain vast limestone exposures. Physicochemical conditions across caves, such as microclimate, humidity, airflow, pH, and light availability, vary with depth and cave origin, creating ecological zones ranging from illuminated entrances to perpetually dark, nutrient‐poor interiors (Turrini et al., 2024). These environmental gradients support complex subaerial biofilms (SABs) composed of phototrophs (e.g., cyanobacteria, green algae) near lighted zones and chemoautotrophs, heterotrophs, and fungi deeper within. Such multi‐kingdom assemblages contribute to mineral precipitation, weathering, and biogeochemical cycling—processes critical to both cave geomorphology and microbial ecology (Zhu et al., 2022).

In contrast to most of the terrestrial environments, subterranean environments provide a more stable microclimate where unique populations can establish and develop specific adaptations. Subaerial biofilms that develop in caves can be non‐phototrophic—usually formed by heterotrophic bacteria, fungi, and protozoa—or phototrophic. Phototrophic cave biofilms can be further subdivided based on whether they developed under natural or artificial light (Gorbushina & Broughton, 2009; Hershey & Barton, 2018). Those developed under artificial light can often be observed in show caves where artificial light fosters a so‐called lampenflora dominated by green algae, diatoms, and cyanobacteria (Mulec, 2019). Whereas non‐phototrophic biofilms in caves are often inconspicuous to the naked eye in terms of color and structure, phototrophic biofilms can be remarkable (Albertano, 2012; Popović et al., 2020; Roldán & Hernández‐Mariné, 2009).

It has, for example, been shown that such phototrophic SABs in caves can exhibit stratification wherein the different organisms such as cyanobacteria, chlorophytes, heterotrophic bacteria/fungi, and mosses are arranged in layers, sometimes reaching several centimeters in thickness (Roldán & Hernández‐Mariné, 2009). Due to these dense organic layers, fungi are often integral parts of the SABs in caves and usually function as decomposers (Jurado et al., 2010), structurally contributing to the biofilms via their fungal hyphae (Popović et al., 2015). Amoeba and diatoms can also be parts of cave biofilms, but cyanobacteria are often the major players (Falasco et al., 2014; García Sánchez et al., 2013).

Cyanobacteria are photosynthetic microorganisms that have been observed in a wide range of environments, from aquatic to terrestrial ecosystems (Büdel, 2024). They are known for their ability to participate in biofilm formation, often as pioneer organisms. These biofilms are complex communities of microorganisms encased in a self‐produced extracellular matrix (Rossi & De Philippis, 2015). When cyanobacteria are the dominant taxa in the biofilm, this matrix is often formed of extracellular polymeric substances (EPS) secreted, for the most, part by the cyanobacteria themselves (Bozan et al., 2024). This EPS matrix has been detected to be the base for the co‐colonization of other life forms such as heterotrophic bacteria, fungi, or protists feeding on the EPS compounds, which results in highly complex, cross‐phylum assemblages (Couradeau et al., 2019; Nelson & Garcia‐Pichel, 2021).

One of the oldest living representatives of the Cyanobacteria, often observed in subaerial biofilms of caves, is the genus *Gloeobacter* (Mareš et al., 2013); members such as *G. violaceus* (Montejano et al., 2018), *G. kilaueensis* (Saw et al., 2013), or *G. morelensis* (Saw et al., 2021) can create dense, slimy, and bright pink biofilms in caves. This genus probably evolved about 3.4 billion years ago (Sánchez‐Baracaldo & Cardona, 2020) and shows structural differences compared to most other members of the phylum, such as the lack of thylakoid membranes (Mareš et al., 2013). Other prominent cave cyanobacteria are *Geitleria* spp., (Davis & Rands, 1981; Friedmann, 1979; Kilgore et al., 2018) *Scytonema julianum* (Aboal et al., 1994; Hoffmann, 1992; Lamprinou, 2012), and *Iphianassa zackieohae* (Panou & Gkelis, 2022), which are known to produce tubular sheaths around their filaments made of calcium carbonate. The ecological role of these structures is under debate (Jung et al., 2024), but functions such as advantages during light trapping, detoxification, or protection from predators have been discussed (Merz‐Preiß, 2000; Pentecost, 1991; Riding, 1991). The erected, comparably large filaments and calcified sheaths of these structures further extend the structure of the overall biofilm into tridimensional space allowing the establishment of a whole micro‐ecosystem including different micro‐niches.

Questions regarding the diversity of phototrophic SABs in caves have mostly been answered by metabarcoding, resulting in large datasets of short DNA fragments that provide an almost complete picture of the diversity but with low taxonomic resolution (Addesso et al., 2024; Pfendler et al., 2018; Turrini et al., 2020). In contrast, studies using isolation techniques investigating the culturable microbiome of cave biofilms are rare (Urzì et al., 2010), and even when this approach has been used, it has been applied to single taxonomic groups (Limrujiwat et al., 2022; Suetrong et al., 2023; Zammit et al., 2011) or based only on microscopic observations (Jakovljević et al., 2024). For this reason, this study aimed to shed light on the culturable microbiome of cyanobacteria‐dominated SABs from a cave in Northern Spain, with a focus on cyanobacteria, green algae, and the associated fungi. Moreover, we hypothesize that very specialized cyanobacterial communities are often located in caves due to niche specialization, whereas fungi originate from the direct environment outside of the cave.

## MATERIALS AND METHODS

### Sampling location and sampling

Sampling took place during June 2022 in a restricted natural cave in Northern Spain, in the vicinity of Bilbao (43.254133, −3.610290). To avoid human influence on the biofilms, we have refrained from adding detailed information on the exact location.

**FIGURE 1:**
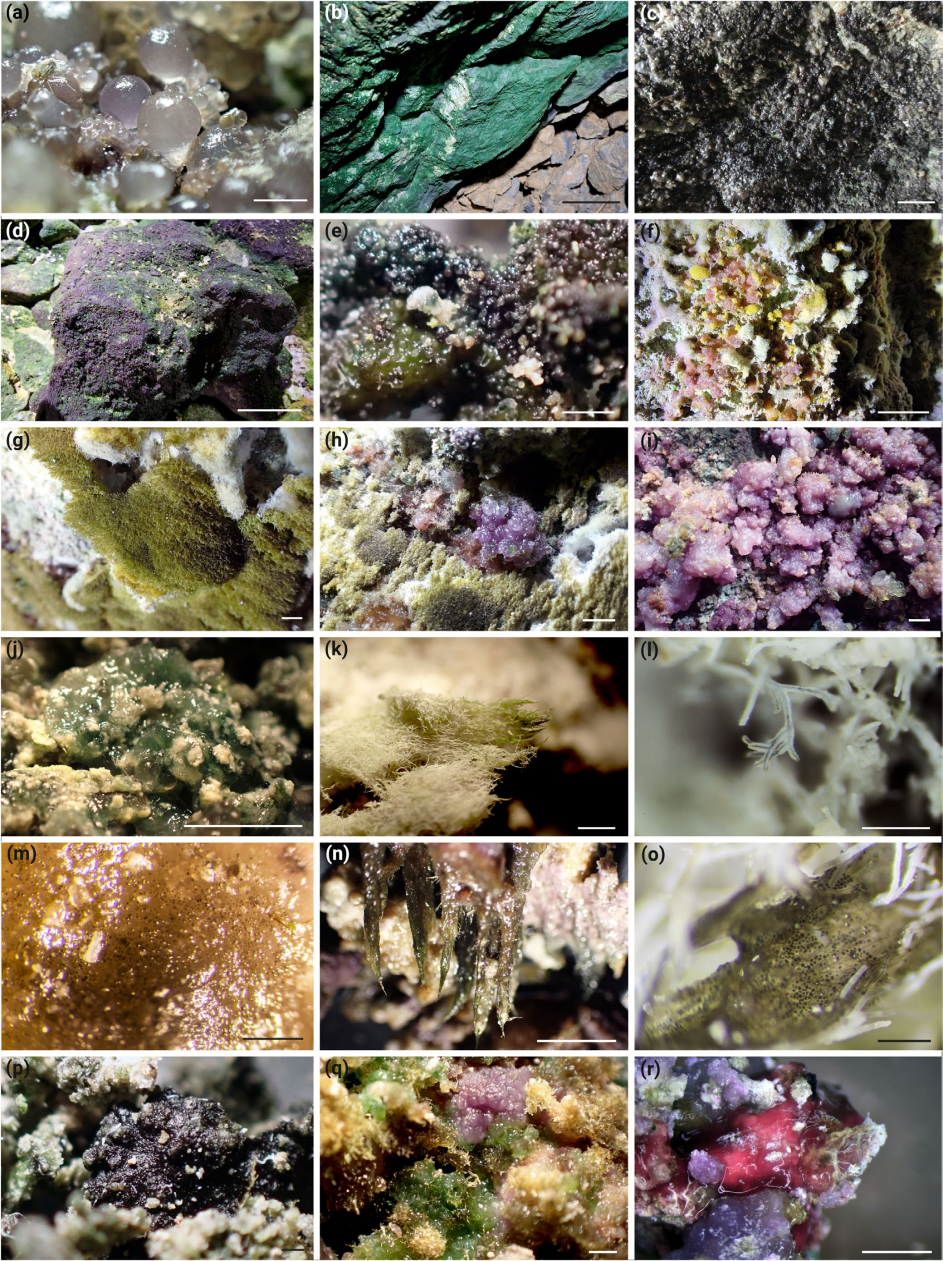
Photographs of cyanobacteria‐dominated cave biofilms. (a) *Nostoc* sp. spheres. (b) *Picosynechococcus* sp. dominated blue‐green biofilm on stones. (c) *Aphanothece* spp. dominated blackish biofilm. (d) Purple biofilm on stones dominated by *Timaviella* sp. and Oculatellales cyanobacteria. (e) Biofilm dominated by different *Nostoc* species. (f) Mixed biofilm. (g) Olive green patches of *Stigonema* sp. (h, i) Purple biofilms of *Gloeobacter* sp. (j) Biofilm dominated by *Chalicogloea cavernicola* associated with calcification. (k) Calcified trichomes of *Scytonema julianum*. (l) Calcified and dichotomously branched trichomes of *Geitleria* sp. (m) Chroococcalean cyanobacteria embedded in extracellular polymeric substances. (n) Cones made of a Nostocalean cyanobacterium. (o) Chroococcalean cyanobacteria embedded in extracellular polymeric substances with calcified trichomes of *Geitleria* sp. (p) *Aphanothece* spp. dominated blackish biofilm. (q) Mixed biofilm made of *Gloeobacter* sp. (purple), cf. *Schizothrix* sp. (green) and *Stigonema* sp. (whitish). (r) Purple biofilms of *Gloeobacter* sp. Scale bar 1 cm; (b, d, f) 10 cm.

Samples of the SABs (Figure 1) were selectively taken from the karstic side walls of the cave at 1.60 m height from the entrance and continuing deeper into the cave by pressing sterile plastic tubes of different volumes (2, 15, 50 mL) directly into the cyanobacteria‐dominated biofilms. The material was then lifted with a sterile spatula and stored in the closed tubes and protected from light at ambient temperature. The distance from the cave entrance was noted for each sample. Directly after transportation the samples were used for microphotography and the cultivation approach.

### Cultivation of biofilm forming eukaryotic green algae, cyanobacteria, and fungi

To identify biofilm‐forming photosynthetic and non‐photosynthetic organisms, an integrative approach was applied. Small fragments of the SABs were picked with sterile tweezers and transferred to 6‐cm agar plates filled with (i) 0.9% solidified BG11 (Stanier et al., 1971) to promote the growth of cyanobacteria, (ii) 0.9% solidified Bold's Basal Medium (BBM; Bischoff & Bold, 1963) for eukaryotic green algae, and (iii) 1% Sabouraud agar for fungi (2% glucose and chloramphenicol; Sifin Diagnostics GmbH, Berlin, Germany).

All enrichment cultures were incubated at 25°C in a culture cabinet at 30 μmol photons · m^−2^ · s^−1^ (CLF PlantClimatics GmbH, Wertingen, Germany) and a 16:8 h light:dark cycle.

After 4 weeks, single colonies of algae or cyanobacteria were transferred to new BBM or BG11 agar plates to establish pure clonal cultures. This process was repeated over a 6‐month period every few weeks until unialgal green algal and cyanobacterial isolates or axenic fungal isolates were generated. The final algal isolates were established as living strains in the research culture collection of the University of Applied Sciences Kaiserslautern (Pirmasens, Germany).

Plates with fungi were inspected 24 hours after incubation and then daily until no new colonies were observed, and single colonies were transferred to fresh Sabouraud agar. Fungal isolates were cultivated on sterile filter paper placed on solidified Sabouraud medium and were removed after 1 week of growth, dried at room temperature in sterile plastic boxes, and stored in the dark.

### Morphological and functional characterization

A digital 3D 4K stereo microscope (VHX‐7000, Keyence Deutschland GmbH, Neu‐Isenburg, Germany) with 40× and 100× magnifications was used to capture images during the growth of the algal isolates on agar plates after 6 weeks. Images of isolated fungi were taken 5 days after transfer to new agar plates with a digital camera (TZ 91, Leica, Wetzlar, Germany). In addition, light microscopy and differential interference contrast microscopy were applied to take detailed images of the isolated algae using a BX51 (Olympus, Tokyo, Japan) coupled with a camera (MicroLive, Bremen, Germany) and MicroLive 5 software (MicroLive, Bremen, Germany) at 40× and 100× magnifications.

Morphological identification of eukaryotic algae was based on Ettl and Gartner (2013), whereas morphological identification of cyanobacteria was based on Komárek and Anagnostidis (2007) and Strunecký et al. (2023) as well as the latest scientific literature on specific taxa.

### Molecular characterization

DNA from the isolates was extracted using the polymerase chain reaction (PCR) protocol as described in Jung et al. (2024). During PCR, the almost complete 16S rRNA gene of the cyanobacteria was amplified (1200 bp) using the primers B2 and B6 (Boyer et al., 2001). Filaments from *Geitleria calcarea* and *Stigonema* sp. were picked for a direct PCR approach as described in Jung et al. (2024) using the same methodology because they could not be isolated. For green algae the primers EAF3 (Marín, 2003) and G800R (Darienko et al., 2019) were used to amplify the SSU (800 bp) rRNA gene. The primers ITS1f (Gardes & Bruns, 1993) and LR3 (Friedl & Rokitta, 1997) covering the partial 18S rRNA, ITS1 and 5.8S ITS2, and partial 28S gene regions were used for the biofilm‐associated fungi (1400 bp). All PCRs were conducted following the co‐cycling conditions described by Jung et al. (2024), and successful amplification was checked by means of gel electrophoresis. Subsequently, PCR products were cleaned using the NucleoSpin® Gel and PCR Clean‐up Kit (Macherey‐Nagel, Düren, Germany) and sent to Genewiz (Leipzig, Germany) for Sanger sequencing.

Sequences of the isolates were compared to those from reference strains in NCBI (http://www.ncbi.nlm.nih.gov) using BLASTn queries to find the closest relatives. When possible, we used sequences of authentic strains or strains from public culture collections, on which the formal taxonomic description of a specific species was based. Multiple alignments of nucleotide sequences were prepared using the MUSCLE algorithm in the software MEGA 11 (Tamura et al., 2021). The evolutionary model that was best suited to the database used was selected based on the lowest Akaike information criterion value and calculated in MEGA 11. Phylogenetic trees were constructed using the web server NGPhylogeny.fr (Lemoine et al., 2019), applying the evolutionary model GTR + G + I for all alignments with 500 generations, each to calculate maximum likelihood (ML) bootstraps. In addition, the program MrBayes 3.2.2 (Ronquist & Huelsenbeck, 2003) was used equivalently to calculate Bayesian inference (BI) with 5,000,000 generations. Two of the four runs of the Markov chain Monte Carlo were made simultaneously, with the trees taken every 500 generations. Split frequencies between runs at the end of the calculations were below 0.01. The trees selected before the likelihood rate reached saturation were subsequently rejected. There was no significant difference shown between the BI and ML trees; thus, single ML trees were used as backbones and edited in iTOL (Letunic & Bork, 2021) and visualized using Biorender.com. Branches for which both statistical analyses (bootstrap probabilities of ML and posterior probabilities of BI) resulted in >80% were marked with an asterisk.

### Enzyme assay for fungi

Insights into the enzymatic activity of isolated fungi provides information regarding their metabolic capabilities, revealing their potential ecological roles within the biofilm. Furthermore, the enzymatic profile offers a complementary, functional layer of information, enabling a better understanding of how phylogenetic diversity relates to ecosystem functioning.

The enzymatic activity was measured using the semiquantitative API ZYM test (bioMérieux, Marcy‐l'Étoile, France) for 19 constitutively expressed lipid‐, protein‐, and carbohydrate‐degrading enzymes. The test was applied following the manufacturer's instructions with a suspension of the isolated fungi. Finally, a value of 0–5 was assigned according to the instructions, corresponding to the established colors where 0–2 color intensity corresponds to a negative reaction and 3–5 to a positive reaction.

## RESULTS

### Isolation and phylogeny

We isolated a total of 58 cyanobacterial strains from the cave SABs, which fell into 21 genera/clades; 24 green algal strains divided into 10 genera/clades, and 41 fungal strains fell into 13 genera/clades. Whenever information on morphology, phylogeny, and ecology matched with those of genera and species that had already been described, we applied nomenclatural terminology at the species level. In instances for which evidence suggested certain strains represented novel species or genera, a conservative approach was taken with terminology. This work could not provide the proper evaluation of those strains following the required additional methodologies and concepts for each organism group.

**FIGURE 2:**
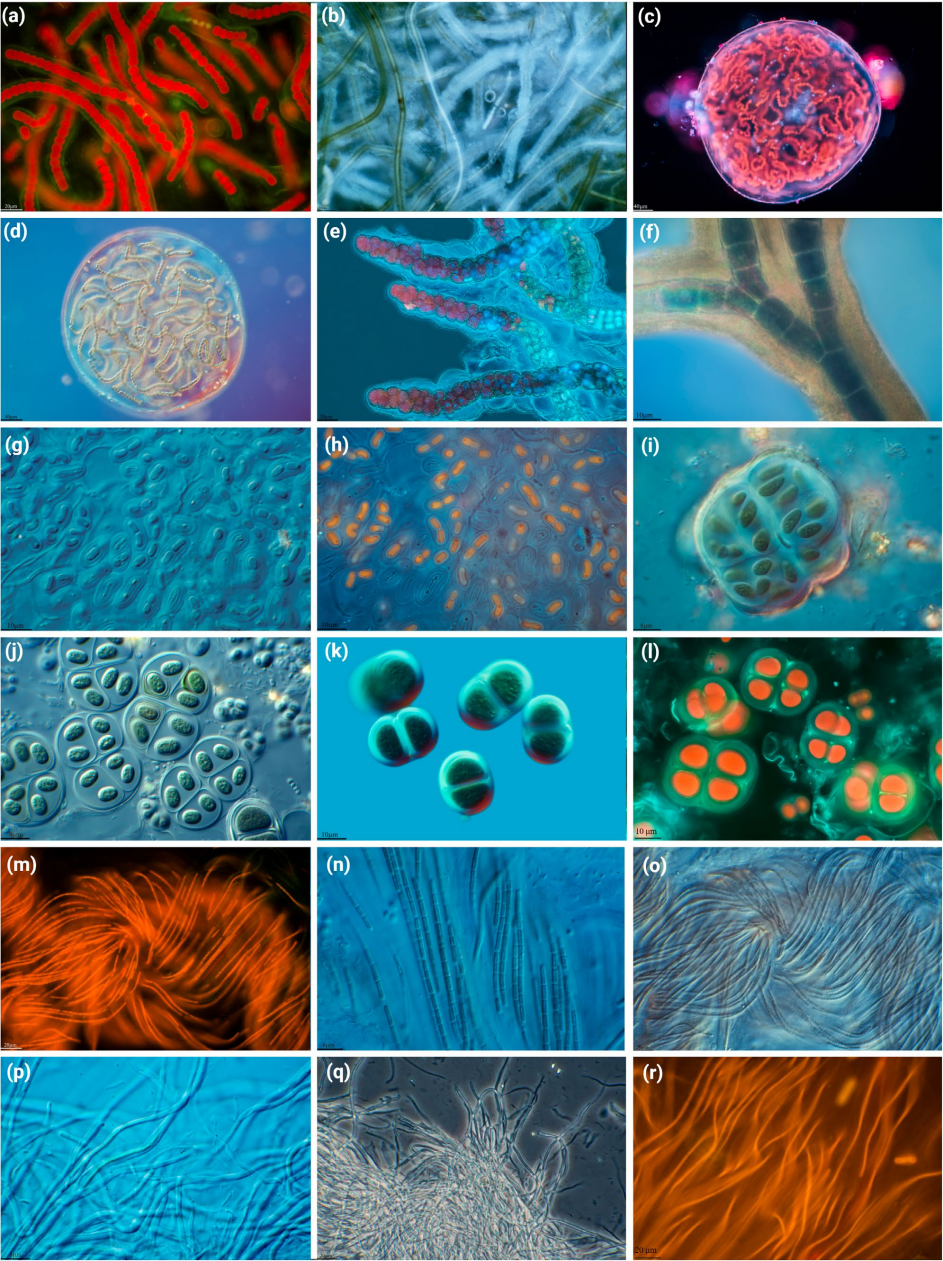
Micrographs of cyanobacteria‐dominated cave biofilms. (a) Nostocalean cyanobacterium from cones. (b) *Scytonema julianum* with calcified sheaths. (c, d) *Nostoc* sp. (e) *Stigonema* sp. (f) *Geitleria* sp. with calcified Y‐trichomes. (g, h) *Gloeobacter* sp. (i) *Aphanothece* sp. (j) *Gloeothece* sp. (k, l) *Limnococcus* sp. (m–r) various filamentous cyanobacteria.

**FIGURE 3:**
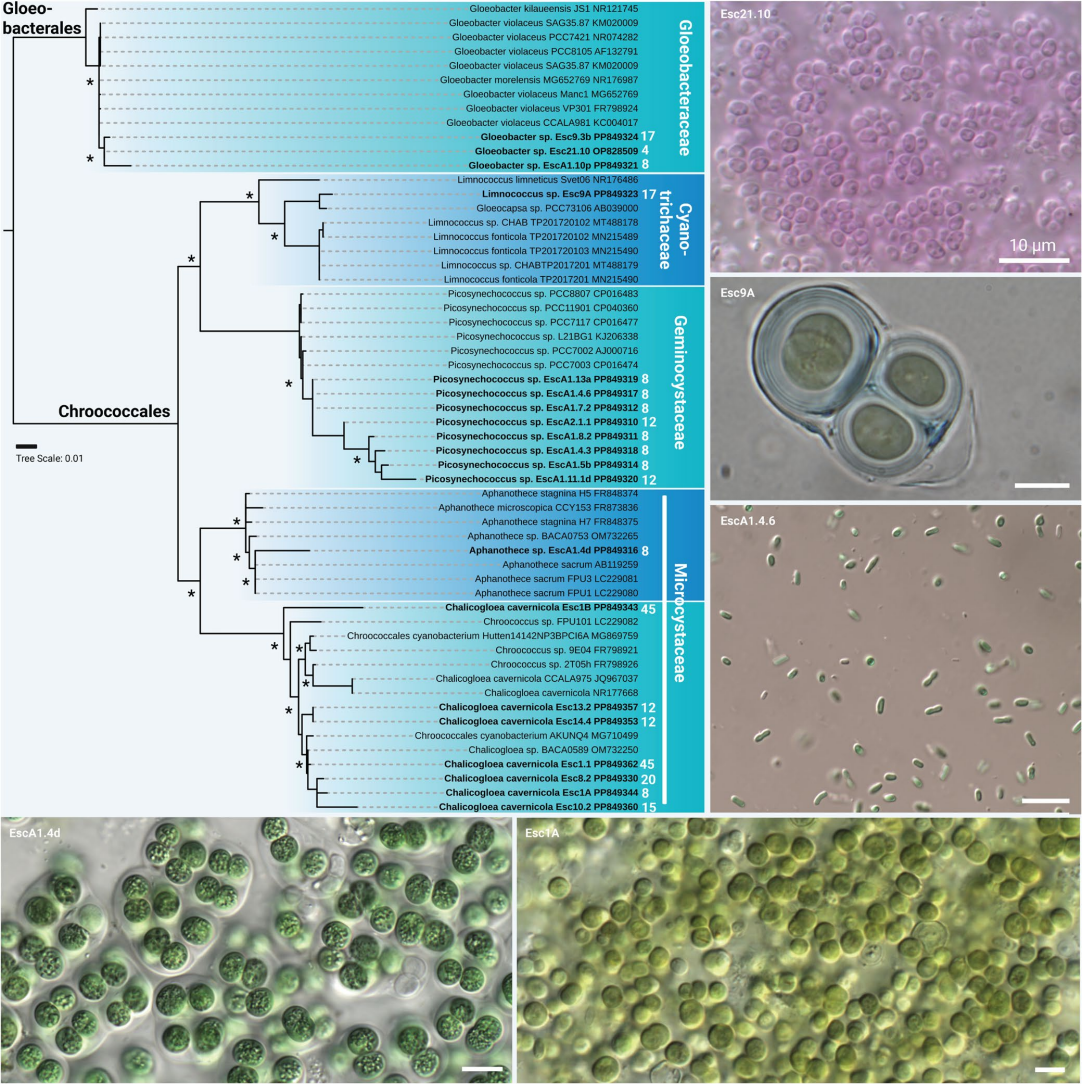
Phylogenetic representation and micrographs of unicellular cyanobacteria. Shown is the full 16S rRNA gene‐derived maximum likelihood (ML) phylogeny of isolated cyanobacteria. Strains in bold are the isolates generated during this study together with their NCBI accession numbers and the distance in meters from the cave entrance is given in white numbers. Nodes with >85% support from ML and Bayesian inference are marked with an asterisk, and microscopic images are shown at the bottom and on the right. Scale bar 10 μm.

**FIGURE 4:**
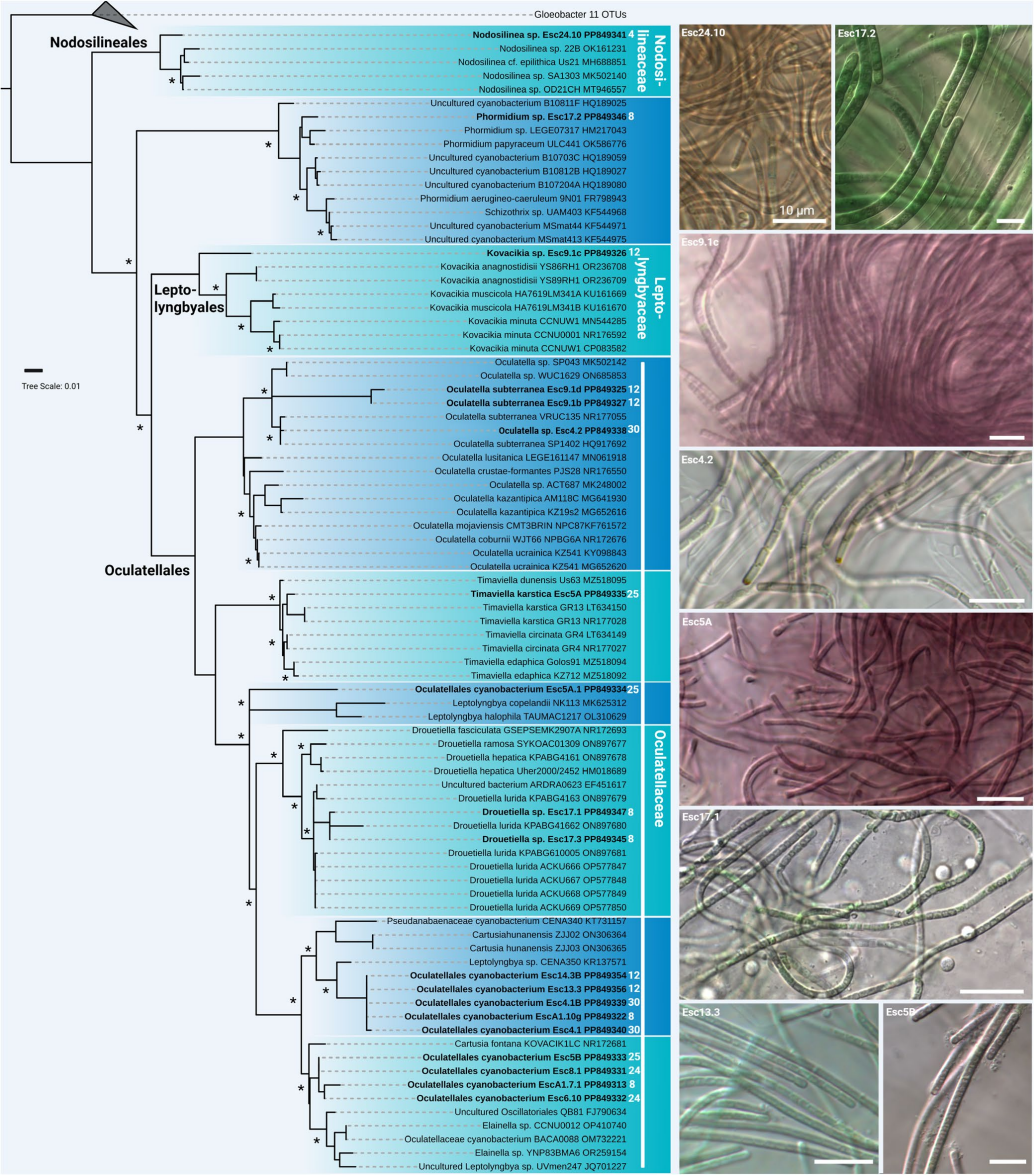
Phylogenetic representation and micrographs of filamentous cyanobacteria. Shown is the full 16S rRNA gene‐derived maximum likelihood (ML) phylogeny of isolated cyanobacteria. Strains in bold are the isolates generated during this study together with their NCBI accession numbers and the distance in meters from the cave entrance is given in white numbers. Nodes with >85% support from ML and Bayesian inference are marked with an asterisk, and microscopic images are shown on the right side. Scale bar 10 μm.

Cyanobacteria representing non‐heterocytous filamentous, unicellular, and heterocytous morphologies were isolated from cave walls at various distances from the entrance (Figure 1). Due to the selective sampling method, we only have referred to the sampling distances from the cave entrance in the figures; future studies will tackle community compositions related to light levels. Unicellular strains were assigned to the genera *Gloeobacter*, *Limnococcus*, *Picosynechoccus*, *Aphanothece*, and *Chalicogloea* (Figures 2g–l and 4). These included, for example, a number of *Picosynechoccus* strains from homogeneous blue‐green biofilms on stones (Figure 1b). Purple biofilms were often created by *Gloeobacter* sp. (Figure 1j) with polar grains and a lacking thylakoid membrane (Figure 2g,h), whereas *Limnococcus* sp. cells surrounded by stratified EPS sheaths were often associated with calcified material (Figure 2m,o). The phylogenies of these three unicellular taxa indicated unique or separated clusters pointing toward new species, and additional unicellular isolates could be assigned to the genus *Aphanothece* sp. that had several cells encapsulated in a limited EPS sheath and *Chalicogloea cavernicola* (Figures 2i,j and 3).

Filamentous strains without heterocytes were assigned to the genera *Nodosilinea*, *Phormidium*, *Kovacikia*, *Oculatella*, *Timaviella*, *Droutiella*, and three unresolved Oculatellales genera (Figure 4). Strains isolated from dense, burgundy mats on stones near the walls, for example, were dominated by *Timaviella karstica* (Figure 1d) whereas a broad range of strains of Oculatellales cyanobacteria representing novel species were isolated and identified from almost all samples (Figure 4). We also identified three strains belonging to *Oculatella* cf. *subterranea*, which could also be identified based on the characteristic orange eye spot in the apical cell (Figure 4).

**FIGURE 5:**
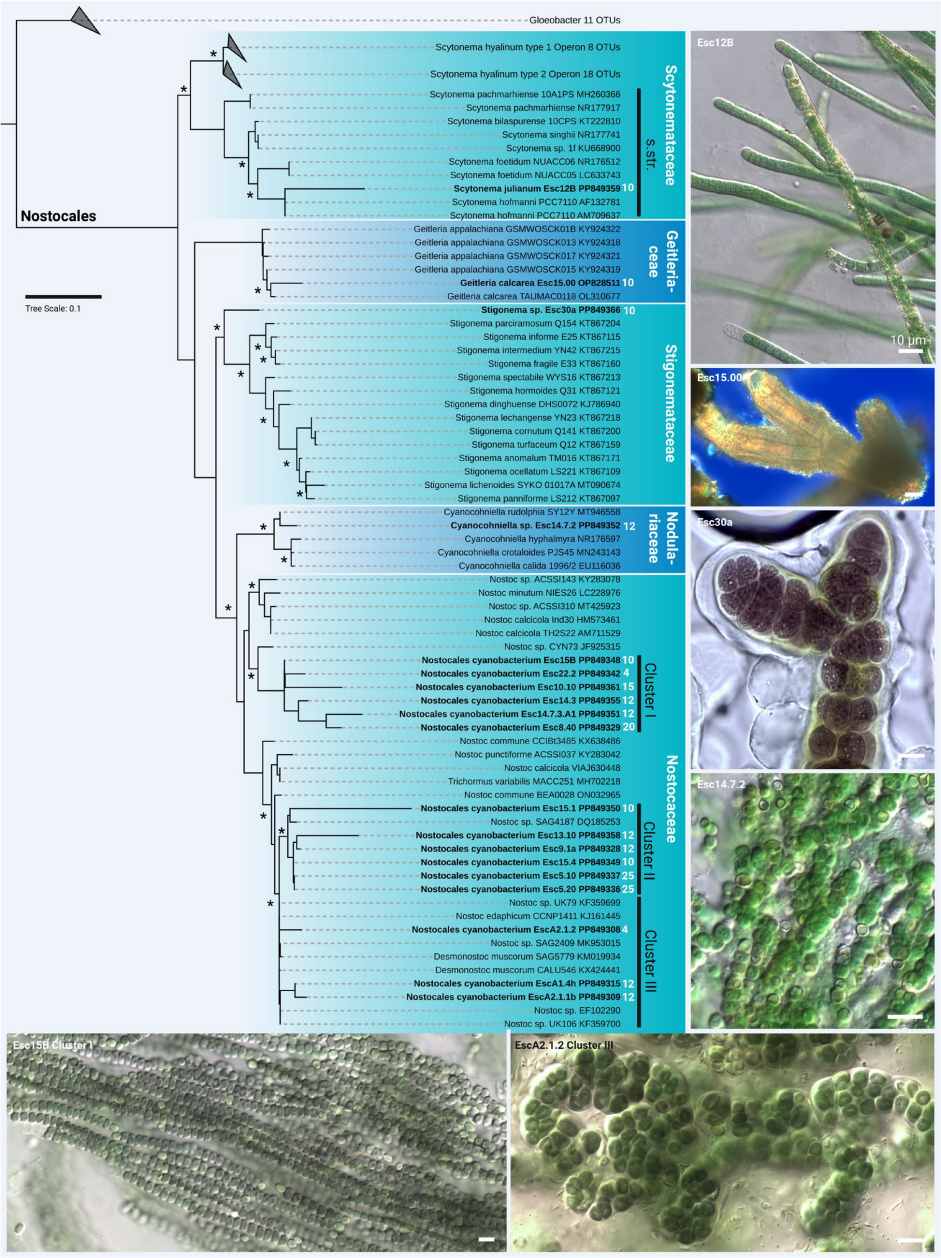
Phylogenetic representation and micrographs of heterocytous cyanobacteria. Shown is the full 16S rRNA gene‐derived maximum likelihood (ML) phylogeny of isolated cyanobacteria. Strains in bold are the isolates generated during this study together with their NCBI accession numbers and the distance in meters from the cave entrance is given in white numbers. Nodes with >85% support from ML and Bayesian inference are marked with an asterisk, and microscopic images are shown at the bottom and on the right side. Scale bar 10 μm.

Strains with heterocytes were assigned to the genera *Scytonema*, *Geitleria*, *Stigonema*, *Cyanocohniella*, and three well‐separated clusters of *Nostoc*‐like strains, which probably represented novel genera or species due to their phylogenetic placements (Figure 5). Rounded, gelatinous balls, for example, were formed on the cave walls by strains of *Nostoc* cluster I and II (Figures 1a and 5), whereas those joining cluster III were inconspicuously associated with other samples (Figures 1e and 5). *Geitleria calcarea* formed whitish tubular structures made of calcium carbonate (Figure 1l) and could not be isolated. Instead, the DNA sequence was generated based on a direct PCR approach, which was also the case for *Stigonema* sp., identified by its multiseriate, true‐branching pattern, which was often growing underneath the cushions formed by *Geitleria calcarea* (Figures 1g and 2e,f). Filaments of calcified sheaths of *Scytonema julianum*, with its false‐branching morphology, were also observed among these cushions and formed additional single patches made of dense, erected, olive‐green filaments (Figures 1k and 2b).

**FIGURE 6:**
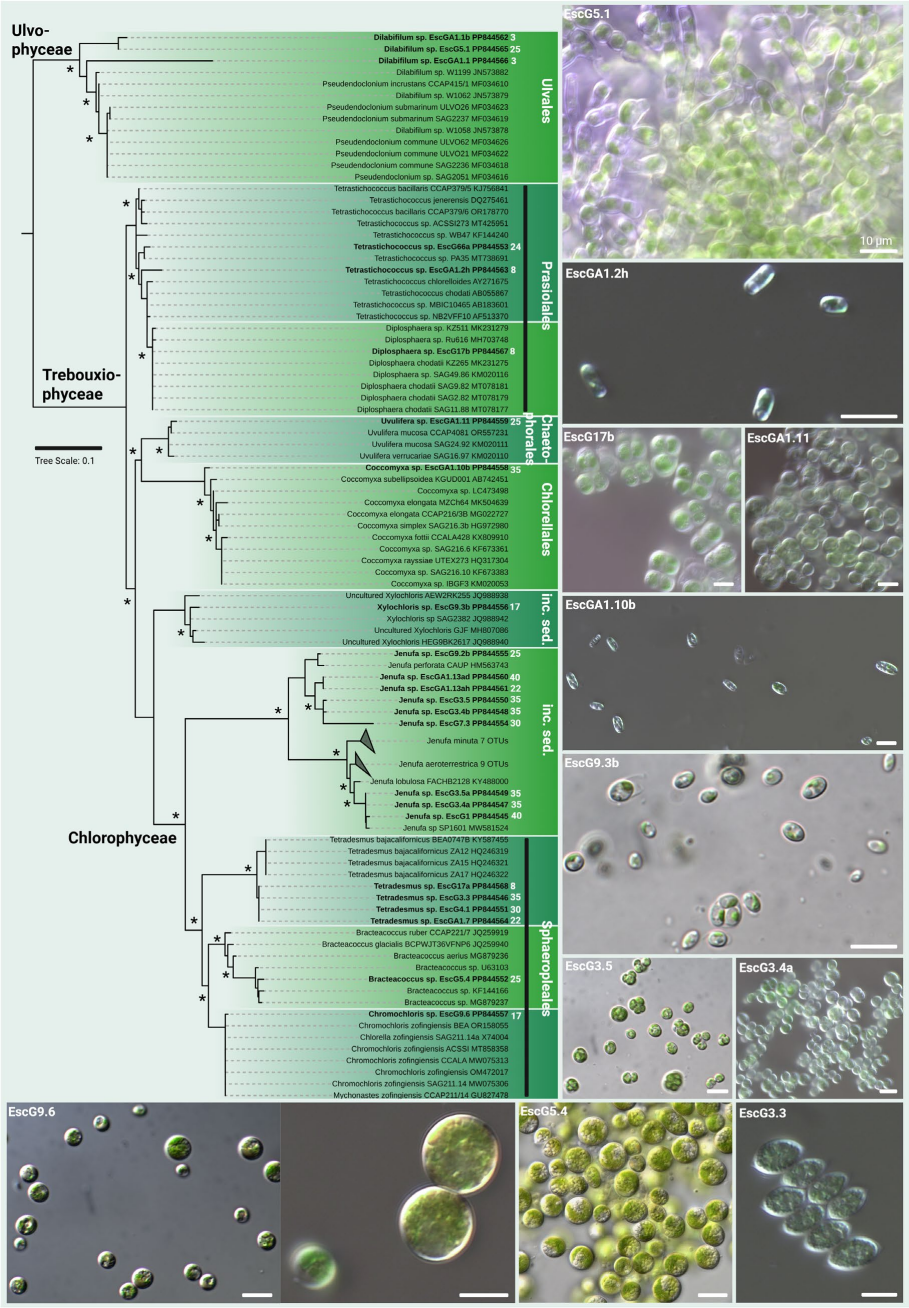
Phylogenetic representation and micrographs of green algae. Shown is the SSU gene‐derived maximum likelihood (ML) phylogeny of isolated green algae. Strains in bold are the isolates generated during this study together with their NCBI accession numbers and the distance in meters from the cave entrance is given in white numbers. Nodes with >85% support from ML and Bayesian inference are marked with an asterisk, and microscopic images are shown at the bottom and on the right side. Scale bar 10 μm.

Green algae fell into the genera *Dilabifilum*, *Tetrastichococcus*, *Diplosphaera*, *Uvulifera*, *Coccomyxa*, *Xylochloris*, *Jenufa*, *Tetradesmus*, *Bracteacoccus*, and *Chromochloris* (Figure 6). Most of the filamentous strains assigned to *Dilabifilum* were isolated from bright greenish patches near the cave entrance, whereas most of the other isolates were mixed with different samples of the cave SABs. Six strains were assigned to the *Jenufa perforata* clade while three additional strains were assigned to the *Jenufa lobulosa* clade based on well‐supported bootstraps. One *Chromochloris* strain was isolated and was supported by its morphology and a strong orange coloration during starvation (Figure 6).

**FIGURE 7:**
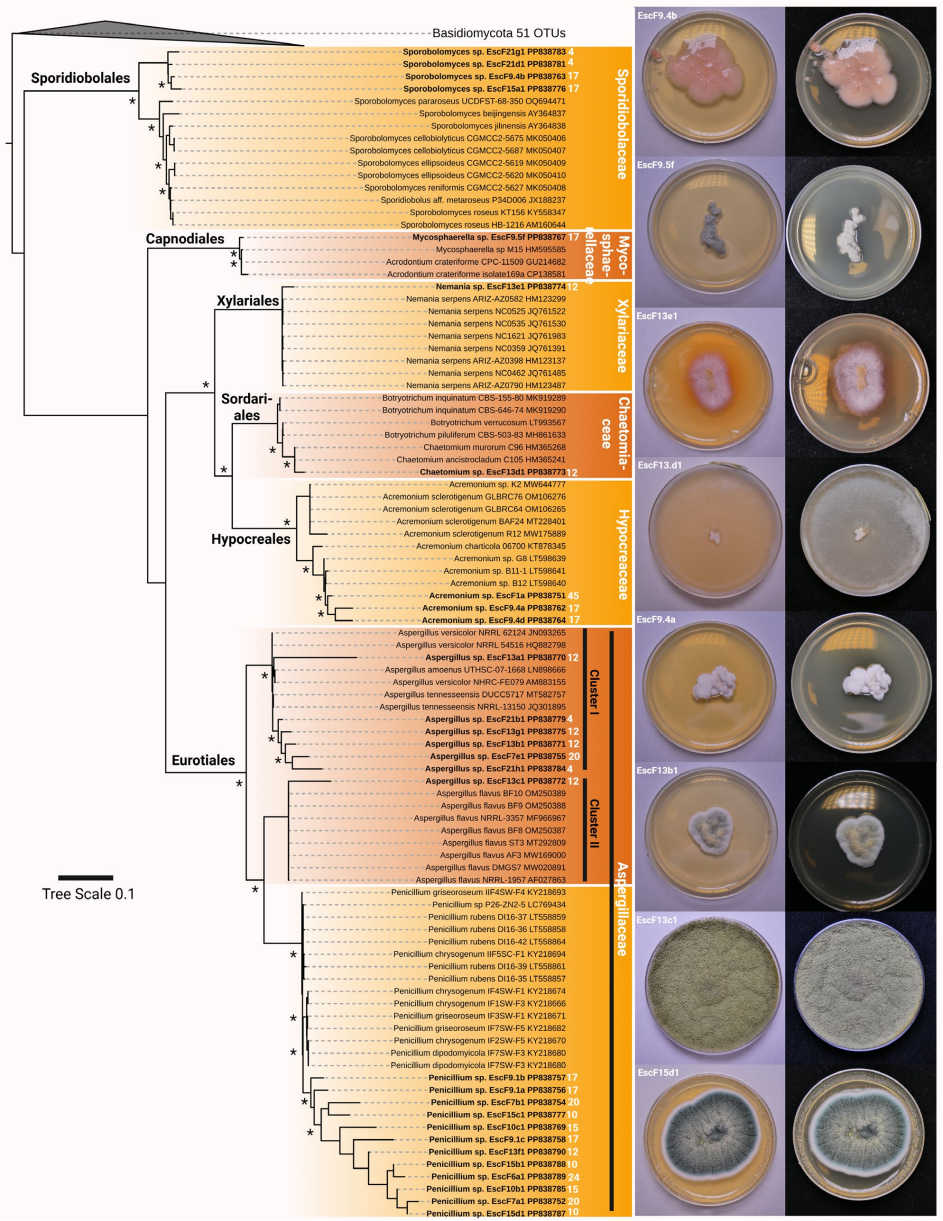
Phylogenetic representation and photographs of Ascomycota. Shown is the ITS1 rRNA region‐derived maximum likelihood (ML) phylogeny of isolated Ascomycota. Strains in bold are the isolates generated during this study together with their NCBI accession numbers and the distance in meters from the cave entrance is given in white numbers. Nodes with >85% support from ML and Bayesian inference are marked with an asterisk, and photographs after 5 days of growth on Sabouraud agar are shown at the bottom and on the right side.

**FIGURE 8:**
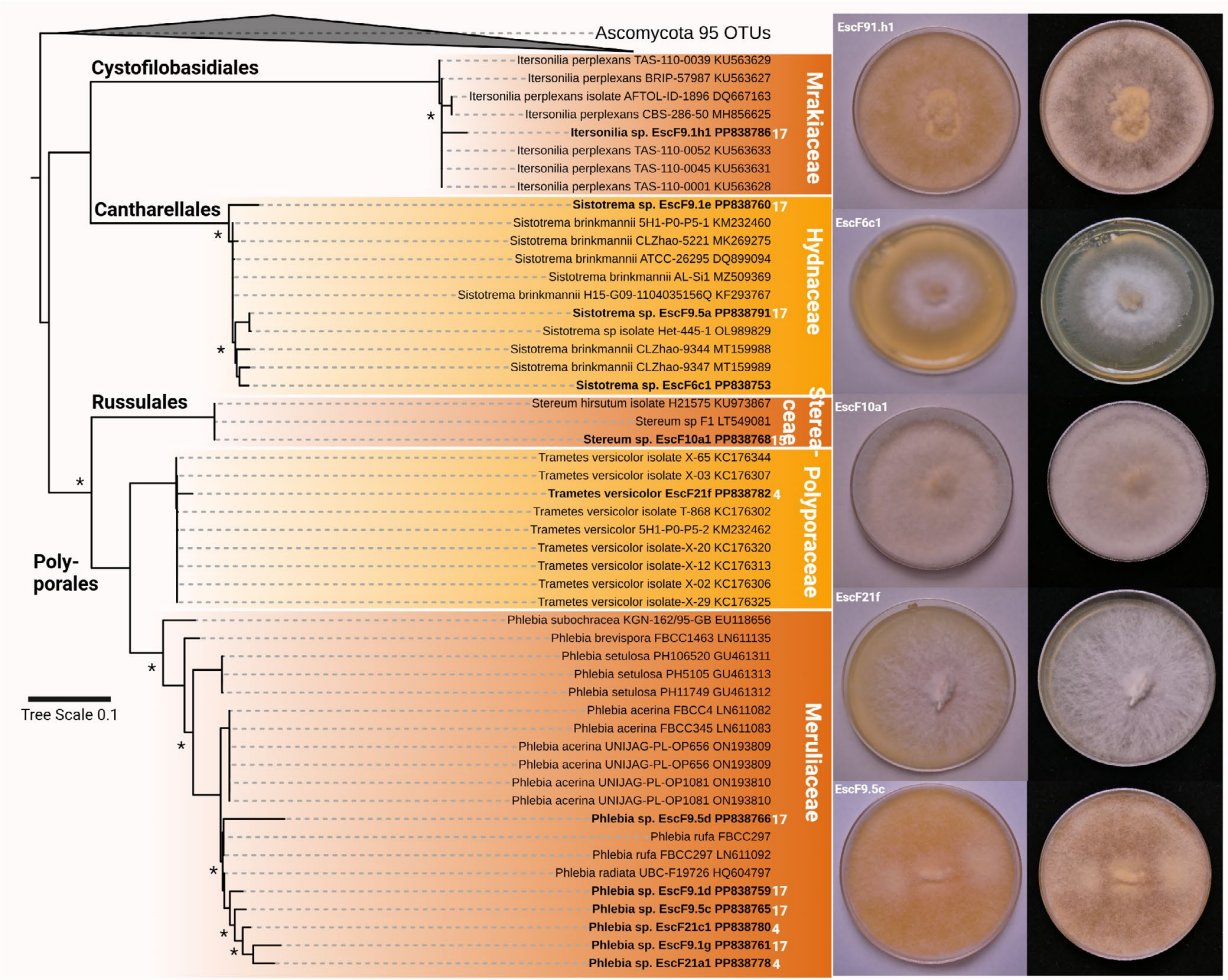
Phylogenetic representation and photographs of Basidiomycota. Shown is the ITS1 rRNA region‐derived maximum likelihood (ML) phylogeny of isolated Basidiomycota. Strains in bold are the isolates generated during this study together with their NCBI accession numbers and the distance in meters from the cave entrance is given in white numbers. Nodes with >85% support from ML and Bayesian inference are marked with an asterisk, and photographs after 5 days of growth on Sabouraud agar are shown at the bottom and on the right side.

The isolated fungi fell into the Ascomycota genera *Sporobolomyces* (mycoparasite, saprophyte), *Mycosphaerella* (phytopathogen), *Nemania* (saprophyte, phytopathogen), *Chaetomium* (saprophyte, phytopathogen, endophyte), *Acremonium* (endophyte, saprophyte), *Penicillium* (saprophyte, endophyte, phytopathogen), and two different clusters of *Aspergillus* (saprophyte, phytopathogen; Figure 7). Basidiomycota fell into the genera *Trametes* (saprophyte, phytopathogen), *Stereum* (phytopathogen, saprophyte), *Itersonilia* (phytopathogen), *Sistotrema*, and *Phlebia* (phytopathogen; Figure 8).

### Fungal enzyme activity

All isolated fungi showed enzymatic activity, and most of them developed taxon‐specific activities (Table 1). Most fungal isolates showed the activity of the following enzymes: (i) esterase (C4; hydrolysis of ester bonds present in lipids; symbiotic and pathogenic interactions), (ii) acid phosphatase (catalyzation of hydrolysis of phosphate groups from different molecules under acidic conditions; nutrient acquisition), (iii) naphtol‐AS‐BI‐phosphohydrolase (hydrolysis of phosphoric acid esters under alkaline conditions; nutrient acquisition, symbiotic and pathogenic interactions), (iv) α‐galactosidase (hydrolysis of α‐galactosidic linkages in various carbohydrates; symbiotic and pathogenic interactions), (v) β‐galactosidase (hydrolysis of glycosidic bonds in various carbohydrates; symbiotic and pathogenic interactions), (vi) β‐glucosidase (hydrolysis of glycosidic bonds in various carbohydrates; cellulose degradation, plant‐fungal interactions, nutrient acquisition), and (vii) N‐acetyl‐β‐glucosaminidase (hydrolysis of N‐acetylglucosamine—GlcNAc—residues from amino sugars and complex carbohydrates; chitin degradation, symbiotic and pathogenic interactions).

**TABLE 1:** Fungal isolates and enzyme test results.

Fungal strain	Enzymes																			
	01	02	03	04	05	06	07	08	09	10	11	12	13	14	15	16	17	18	19	20
*Acremonium* sp. EscF1a			y								y	y	y					y		
*Acremonium* sp. EscF9.4a			y								y	y	y					y		
*Acremonium* sp. EscF9.4d			y								y	y	y					y		
*Sporobolomyces* sp. EscF9.4b			y	y		y					y	y								
*Sporobolomyces* sp. EscF15a1		y	y	y		y	y				y	y	y	y	y	y	y	y		
*Sporobolomyces* sp. EscF21g1			y	y		y	y				y	y								
*Sporobolomyces* sp. EscF21d1			y	y		y					y	y								
*Penicillium* sp. EscF15b1		y	y	y							y		y	y		y	y	y		
*Penicillium* sp. EscF9.1c			y								y	y	y	y			y	y		
*Penicillium* sp. EscF15c1		y									y	y	y				y	y		
*Penicillium* sp. EscF13f1			y								y	y	y	y			y	y		
*Penicillium* sp. EscF9.1b											y	y		y			y	y		
*Penicillium* sp. EscF9.1a											y	y		y			y	y		
*Penicillium* sp. EscF7b1											y	y		y			y	y		
*Penicillium* sp. EscF10c1											y	y		y			y	y		
*Penicillium* sp. EscF6a1											y	y		y			y	y		
*Penicillium* sp. EscF10b1											y	y		y			y	y		
*Penicillium* sp. EscF15d1											y	y		y			y	y		
*Penicillium* sp. EscF7a1											y	y		y			y	y		
*Stereum* sp. EscF10a1			y								y	y	y	y	y	y	y	y	y	
*Mycosphaerella* sp. EscF9.5f			y								y									
*Itersonilla* sp. EscF9.1h1			y	y							y						y	y		
*Sistotrema* sp. EscF6c1											y									
*Sistotrema* sp. EscF9.5a											y									
*Sistotrema* sp. EscF9.1e											y									
*Aspergillus* sp. EscF13g1			y								y						y	y		
*Aspergillus* sp. EscF13c1		y	y								y			y			y	y		
*Aspergillus* sp. EscF13a1			y								y	y	y	y			y	y		
*Aspergillus* sp. EscF13b1											y	y	y	y			y	y		
*Aspergillus* sp. EscF7e1		y	y								y						y	y		
*Aspergillus* sp. EscF21b1			y								y	y	y				y	y		
*Aspergillus* sp. EscF21h1		y	y								y			y			y	y		
*Nemania* sp. EscF13e1											y						y			
*Phlebia* sp. EscF9.5d		y	y	y							y	y	y	y	y	y	y	y		y
*Phlebia* sp. EscF9.1d		y	y	y							y	y	y	y	y	y	y	y		y
*Phlebia* sp. EscF9.5c		y	y	y							y	y	y	y	y	y	y	y		y
*Phlebia* sp. EscF9.1g		y	y	y							y	y	y	y	y	y	y	y		y
*Phlebia* sp. EscF21c1		y	y	y							y	y	y	y	y	y	y	y		y
*Phlebia* sp. EscF21a1		y	y	y							y	y	y	y	y	y	y	y		
*Trametes versicolor* EscF21f											y	y		y	y					
*Chaetomium* sp. EscF13d1		y															y			

*Note*: Tested were the following enzymes: 1, control; 2, alkaline phosphatase; 3, esterase (C4); 4, esterase lipase (C8); 5, lipase (C14); 6, leucine arylamidase; 7, valine arylamidase; 8, cystine arylamidase; 9, trypsin; 10, α‐chymotrypsin; 11, acid phosphatase; 12, naphthol‐AS‐BI‐phosphohydrolase; 13, α‐galactosidase; 14, β‐galactosidase; 15, β‐glucoronidase; 16, α‐glucosidase; 17, β‐glucosidase; 18, *N*‐acetyl‐β‐glucosamidase; 19, α‐mannosidase, 20, α‐fucosidase.

None of the fungi isolated tested positive for α‐chymotrypsin (degradation of extracellular proteins from organic matter; pathogenic interactions) nor for acid‐ and alkaline phosphatase (involved in phosphate acquisition) and Naphthanol‐AS‐BI‐phosphohydrolase.

## DISCUSSION

This study has set the framework for additional future studies, enabling the combination of culture‐dependent with culture‐independent methods, allowing deeper investigation into the community composition and structure of cave biofilms in relation to, for example, light levels. Culture‐dependent approaches supported by sequencing of taxonomically relevant gene regions have gained increasing interest, especially for cyanobacteria, for several reasons. First, the phylum recently received a drastic phylogenetic and taxonomic update by Strunecký et al. (2023) in which the phylum was restructured and now comprises 20 orders and 43 families including some internal rearrangements. For example, the former order Pleurocapsales now represents the family Pleurocapsaceae within the order Chroococcales (Strunecký et al., 2023). Second, a curated database for metabarcoding‐derived data from Cyanobacteria called CyanoSeq was created, which constantly integrates the latest taxonomic updates (Lefler et al., 2023). As a result, systematic changes based on formally newly described Cyanobacterial genera and species are incorporated into CyanoSeq by experts, which allows a more accurate assignment of metabarcoding data (Lefler et al., 2023). This decreases underestimations during such studies and allows a corrected assignment of short DNA reads to taxonomic entities on a comprehensive basis (Jung, 2023). Third, the population‐genomics approach applied to cyanobacteria has advanced our understanding of cyanobacterial diversity by moving beyond single‐locus phylogenies to genome‐wide analyses of intraspecific variation (Dvořák et al., 2023). By sequencing multiple strains of the same or closely related cyanobacterial taxa, this approach has enabled the identification of population structure, recombination patterns, and adaptive genetic traits within and across ecological niches. One of the key benefits of this method is its capacity to detect cryptic diversity and delineate species boundaries with higher resolution, thereby supporting a more robust genomic framework for cyanobacterial taxonomy. Crucially, the success of this approach depends on the availability of cultured isolates, which provide high‐quality, complete genome sequences and allow for reproducible phenotypic characterization under controlled conditions. Isolates also make it possible to link genotypic variation to ecological and physiological traits, strengthening evolutionary and ecological interpretations of genomic patterns observed in natural populations.

As a consequence of these developments, culture‐dependent approaches applied to specialized communities, as presented here, will allow an in‐depth characterization of living strains, which will help to update systems relying on community levels. In conclusion, these developments have the potential to enhance the interpretation of entire ecosystems and their microbial communities. These developments also underscore the importance of isolating novel strains and establishing culture collections as a foundation for generating morphological data and conducting DNA sequence comparisons with authentic strains or type strains.

### Cyanobacteria

As a general concept, cave microorganisms can be categorized as resident and non‐resident, where the latter accidentally and/or occasionally enter the cave system by water, wind, or air as spores or are even carried in by animals. Cyanobacteria have been divided into three groups (Hoffmann, 2003): troglobiotic (obligatory cavernicolous organisms that cannot survive outside the cave), troglophilic (organisms growing and reproducing in caves), and trogloxenic (organisms which accidentally appear in the cave). This theory was supported by Lamprinou et al. (2014), who suggested that only a few cyanobacteria were obligatorily bound to the cave environment, such as *Symphyonema cavernicolum*, *Herpyzonema pulverulentum*, *Geitleria floridana*, and *Geitleria calcarea*. The persistent failure to isolate *Geitleria appalachiana* from an American cave (Kilgore et al., 2018) and our repeated unsuccessful attempts to culture *Geitleria* spp. from the cave system investigated in this study highlight the challenges of cultivating certain cave‐dwelling cyanobacteria. Although the concept of troglobiotic organisms—species uniquely adapted to cave environments—provides a useful framework for considering ecological specialization, it alone does not explain the difficulty in cultivating these taxa. Other factors likely contribute, such as unknown physiological requirements, specific biotic interactions, or ecological parameters (e.g., light intensity, temperature fluctuations, humidity, mineral availability) that have not been adequately replicated in laboratory conditions. Further, we did not specifically test a variety of media such as nitrogen‐free BG11 tailored for heterocytous cyanobacteria, which may be required for some taxa such as *Stigonema* or *Syctonema julianum*, nor did we explore the full range of possible light–dark cycles or microaerobic chamber conditions that might more closely mimic cave habitats. Thus, although endemism among cyanobacteria appears to be rare (Durieu et al., 2024), caves may harbor highly specialized lineages (Hershey & Barton, 2018; Rabelo et al., 2018) for which isolation is hindered by a combination of ecological complexity and insufficiently adapted cultivation methods. Previous studies have identified cave cyanobacterial taxa that have either not been isolated or were only observed in cave SABs, such as (for example) *Syctonema julianum* with its calcified sheaths (Jones & Peng, 2014), the unicellular *Chalicogloea cavernicola* (Roldán et al., 2013), *Oculatella subterranea* with the typical eye‐spot (Zammit et al., 2012), *Timaviella karstica*, which has purple filaments (Sciuto et al., 2017), and *Gloeobacter kilauensis* (Saw et al., 2013). Our study, however, successfully isolated members of the aforementioned named taxa, thus providing a valuable resource for future investigations either into their genome‐based phylogenetic relationships and eco‐physiological adaptations or for biotechnology. Future isolation efforts could benefit from systematically testing media formulations lacking combined nitrogen, simulating low‐light or light‐free environments, and incorporating environmental parameters measured directly from cave microhabitats.

### Green algae

Eukaryotic green algae are most often associated with cyanobacteria‐dominated SABs in caves, and their diversity has been examined in a variety of locations (Baković et al., 2022; Vinogradova & Mikhailyuk, 2009). Similar to our results, green algae have been shown to contribute less to species richness compared to cyanobacteria, particularly in natural cave zones beyond the entrance area (Kozlova et al., 2019). However, this pattern may not apply uniformly across all cave habitats. In lampenflora communities, green algae can become more prominent due to enhanced light availability and may exhibit greater species richness and biomass. In contrast, in dimly lit or aphotic zones, their contribution remains limited. Only a few taxa, such as members of the genus *Dilabifilium*, have been reported to grow as isolated patches (Czerwik‐Marcinkowska, 2013), and such occurrences appear to be habitat‐specific. In contrast, more cosmopolitan strains such as *Coccomyxa* sp. have also been isolated and have been reported from a great amplitude of habitats including lakes and terrestrial biofilms or associated with mosses or symbiotic in lichens, but they have also been reported from caves (Jurado et al., 2022; Popović et al., 2023; Sánchez‐España et al., 2020).

This is similar for the genus *Jenufa*, which has been described from sandstone surfaces, walls of buildings, and tree barks (Prochazkova et al., 2015) but has also been reported from caves (Hodač et al., 2012; Jurado et al., 2022). As already speculated by Hodač et al. (2012) and Jurado et al. (2022), the genus *Jenufa* holds a so‐far unknown diversity to which we have contributed some insights into by providing strains falling in at least two sub‐clusters (Figure 6).

Interestingly, we also isolated a strain of the genus *Chromochloris*, which has, so far, been mainly described from soil or tree bark (Wood et al., 2024). *Chromochloris zofingiensis* (Chlorophyceae) is emerging as a model for investigating metabolic flexibility in green algae or astaxanthin production (Roth et al., 2017; Wood et al., 2024). Under specific conditions such as heterotrophy, nitrogen deprivation, or high light, *C. zofingiensis* accumulates high amounts of the valuable secondary ketocarotenoid astaxanthin and biofuel precursors (Zhang et al., 2021). The strain from the cave environment that we isolated might be adapted to low‐light conditions so that astaxanthin production in this strain could be altered compared to the model strains, making it a potential candidate for biotechnological applications.

### Fungi

In general, fungi in caves have been understudied (see Savković et al., 2025 and references therein), and most existing studies have focused on fungi that are not associated with cyanobacterial biofilms (Cunningham et al., 1995; Poli et al., 2024; Vanderwolf et al., 2013). It has been shown, however, that caves represent an interesting environment for fungi, hosting a great diversity that includes many yet undescribed species (Poli et al., 2024). Zhang et al. (2017), for example, isolated 563 fungal isolates assigned to 246 species in 116 genera from two Karst caves in China. Of those species, 59% were newly described from Karst caves, including 20 that were new to science. Certain fungi of the Ascomycetes, such as *Penicillium* spp., *Aspergillus* spp., or *Cladosporium* spp. have frequently been reported from caves during various studies that included both culture‐dependent and culture‐independent approaches (Poli et al., 2024; Popović et al., 2015); these results were also supported during the work presented here. In addition, it has often been highlighted that fungi from caves are mainly trogloxenic or originate from the direct environment outside of the cave (Vanderwolf et al., 2013; Zhang et al., 2017). Our results support this contention, since the direct environment of the cave was a mixed forest, and we isolated a high degree of wood‐decaying fungi such as *Trametes versicolor, Stereum* spp., or *Phlebia* spp. Many strains obtained in this study were known as plant endophytic or pathogenic species, which their enzyme activity profiles supported. These strains may originate from outside environments, dispersed through water or air flow, and remain alive in caves, where they grow based on the organic matter provided by the phototrophic SABs. Kuzmina et al. (2012), for example, suggested that cave systems might be a good harbor for the development and preservation of allochthonous microorganisms, including pathogenic species.

## CONCLUSIONS

This study has provided substantial evidence in support of the hypothesis that specialized microbial communities are often located in caves (e.g., Hershey & Barton, 2018; Rabelo et al., 2018). Through culture‐dependent methods, it was shown that several cyanobacterial taxa such as *Geitleria calcarea*, *Scytonema julianum*, *Chalicogloea cavernicola*, *Oculatella subterranean*, and *Timaviella karstica*, were either associated exclusively or primarily with cave environments. Identification of potentially novel cyanobacterial genera and species, especially within Nostocales and Oculatellales, supported the hypothesis. This evidence suggested caves may act as isolated evolutionary niches. The presence of obligate cave organisms (troglobiotic species) and the repeated failures to culture certain organisms outside of their natural cave habitats further underscore the specialized adaptation of these species. Although the fungi and green algae included many cosmopolitan or trogloxenic species, the overall results highlight caves as unique ecological niches that foster specialization, particularly among cyanobacteria.

## AUTHOR CONTRIBUTIONS

**Patrick Jung:** Conceptualization (lead); data curation (lead); formal analysis (lead); funding acquisition (lead); investigation (lead); methodology (lead); project administration (lead); software (lead); supervision (lead); validation (equal); visualization (lead); writing – original draft (lead). **Laura Briegel‐Williams:** Conceptualization (equal); data curation (equal); writing – original draft (equal); writing – review and editing (equal). **Dennis J. Nürnberg:** Investigation (equal); validation (equal); writing – review and editing (equal). **Tobith Wolf:** Investigation (equal); methodology (equal); writing – review and editing (equal). **Antonio Guillen:** Resources (equal); writing – review and editing (equal). **Manuel Leira:** Resources (equal); writing – review and editing (equal). **Michael Lakatos:** Investigation (equal); supervision (equal); Resources (equal); writing – review and editing (equal).

## FUNDING INFORMATION

The project is funded by the German Research Foundation (DFG) with the grant number JU 3228/1‐1 to PJ and NU 421/1 to DJN. PJ and LBW are funded by the Carl‐Zeiss Foundation (P2023‐03‐051). LBW was also internally funded by HS KL. ML is funded by the Ministry of Science and Health Rhineland‐Palatinate (BioTerrMa, 724‐0079#2024/0004‐1501 15404) and by the Federal Ministry of Education and Research (W2V‐Strategy2Value, 03WIR4502A and Blue2Value 03WIR4508A).
